# Exploration of the pattern, clinical presentation and outcome of children with renal diseases: A 14-year retrospective study at a teaching hospital in Ghana

**DOI:** 10.1371/journal.pone.0321317

**Published:** 2025-04-25

**Authors:** George Nkrumah Osei, Emmanuel Worae, Betty Effie Adoko Ghartey, Perditer Okyere, Felix Abekah Botchway, Sampson K. Djonor, Richard K.D. Ephraim

**Affiliations:** 1 Department of Medical Laboratory Science, School of Allied Health Sciences, University of Cape Coast, Cape Coast, Ghana; 2 Department of Internal Medicine, School of Medical Sciences, College of Health Sciences, Kwame Nkrumah University of Science and Technology, Kumasi, Ghana; 3 Department of Medical Laboratory Technology, Faculty of Applied Sciences, Accra Technical University, Accra, Ghana; 4 Department of Immunology, Laboratory Sub-BMC, Korle-Bu Teaching Hospital, Accra, Ghana; Dilla University College of Health Sciences, ETHIOPIA

## Abstract

**Introduction:**

Paediatric nephropathy, a condition associated with significant morbidity and mortality, is increasing in developing countries. Data on children with renal diseases are insufficient in Ghana despite the risk it poses. This study assessed the pattern, spectrum, outcome, and predictors of dialysis and death among children with renal disease at Korle Bu Teaching Hospital (KBTH), Ghana.

**Materials and methods:**

A cross-sectional retrospective review was conducted among children aged 0–17 years. Demographic characteristics, clinical and laboratory data, information on dialysis and treatment outcomes were obtained from their medical records and analyzed accordingly using STATA software version 15.1.

**Results:**

A total of 332 children with renal diseases were seen (median age of 6 years), with 200(60.98%) being males. The most common renal diseases were nephrotic syndrome 141(42.47%) and acute kidney injury (AKI) 87(26.20%). Idiopathic (unknown) causes (82.98%) and intravascular hemolysis secondary to malaria (41.38%) were the major causes of nephrotic syndrome and acute kidney injury respectively. A death rate of 15.06% resulting mostly from AKI (6.33%) was observed whilst 7.83% underwent dialysis. Predictors of dialysis among those who had dialysis included being a female and having acute on chronic kidney disease whilst having high white blood cell count and acute on chronic kidney disease were significant predictors of death among children with renal diseases.

**Conclusion:**

Paediatric renal diseases at KBTH were dominated by nephrotic syndrome and AKI. Timely treatment and prevention of common infectious agents and conditions causing intravascular hemolysis, which can contribute to paediatric renal diseases in Ghana, is needed to help reduce their progression to various forms of kidney disease.

## Introduction

Paediatric nephrology is not a priority in medicine in developing countries contrary to developed countries in relation to its preventive and curative measures [1]. This is partly due to inadequate data on paediatric renal diseases and a focus on communicable diseases in developing countries [2]. The prevalence of paediatric renal diseases ranges from 2.2% to 8.9% in some developing countries including Nigeria, Ethiopia and Nepal [3–6]. This reveals that renal diseases are common among children in Nigeria, Ethiopia, and possibly other developing countries.

Acute kidney injury (AKI), nephrotic syndrome (NS), acute glomerulonephritis (AGN), and urinary tract infection (UTI) are the most reported renal diseases among children in sub-Saharan Africa [2,3,5–14]. However, the prevalence of CKD among children remains low in sub-Saharan Africa [3,5,8,11,14,15]. The etiology of paediatric renal diseases varies widely according to the clinical setting, geographical region, and age [10]. Infection, hypertension, diarrhoea, severe malaria and herbal concoctions among others have been reported as causes of paediatric renal diseases in sub-Saharan Africa [2,3,6,10,16].

Symptoms specific to renal disease like generalized or facial swelling, haematuria, burning micturition, and oliguria help in early diagnosis and subsequent early intervention to prevent complications [6,17,18]. However, children with renal diseases may exhibit nonspecific signs and symptoms unrelated to the urinary system causing kidney disease to go undetected [5].

Paediatric renal diseases are also associated with significant morbidity and mortality, especially among hospitalized patients [2]. Death rates ranging from 5% to 17.7% have been reported among children with renal diseases and are mostly due to AKI, CKD, and acute glomerulonephritis [2–4,6,15]. A retrospective analysis of 20 years of autopsy records from leading teaching hospitals in Ghana found a mortality rate of 5.9% associated with renal diseases [19]. This projects renal diseases as an important cause of death in Ghana, underscoring the need for a deeper understanding of the pattern, clinical presentation and outcomes of these conditions, particularly among the paediatric population who may be particularly vulnerable. Understanding the epidemiology of paediatric renal diseases is therefore crucial for effective health planning, resource allocation, and provision of quality renal services [3,20]. To help address the issue of increased morbidity and mortality among children with renal diseases coupled with the insufficient data on paediatric renal diseases in Ghana, this study assessed the pattern, spectrum, outcome and predictors of dialysis and death among children with renal diseases at Korle Bu Teaching Hospital (KBTH), Ghana. The findings in this study will also aid efforts to detect renal diseases in their early stages among children for effective diagnosis and management.

## Materials and methods

### Study site, design, population and sample size

The study was conducted at Korle-Bu Teaching Hospital (KBTH). The hospital, the foremost teaching hospital in Ghana and the third largest hospital in Africa, is a major referral clinic for children with renal diseases in the southern part of Ghana. The Child Health Department at Korle-Bu Teaching Hospital sees on average 36,000 paediatric patients visit annually across its various specialty clinics. There are two (2) paediatric nephrologists who attend to cases of renal significance. A retrospective cross-sectional study was conducted to explore the pattern, clinical presentation and outcome of children aged 0–17 years with renal diseases who received treatment at the Korle-Bu Teaching Hospital in Ghana from 2009 to 2022. This study used all data available at the time of data collection.

### Eligibility criteria

Participants with a complete set of information diagnosed within the study period were eligible for the study. Children above 17 years and those who were admitted to the facility before 2009 were excluded.

### Sampling and data collection procedure

A consecutive sampling technique was employed to review and collect data from the medical records of participants. The medical records of each patient were reviewed individually to prevent any potential overcounting of the same patient over the study period. Collected data included demographic characteristics (age, sex), clinical data (types, causes, symptoms of renal disease and duration of clinic attendance), laboratory data (haematological [full blood count], biochemical [kidney function test]), information on dialysis and outcome of treatment (alive, dead). The data was collected from 6th February 2022–23rd June 2022.

### Ethical consideration

The study was approved by the Department of Medical Laboratory Science, University of Cape Coast. The Korle Bu Teaching Hospital Scientific and Technical Committee (KBTH-STC) approved our study protocol. The protocol identification number was KBTH-STC/IRB/000200/2021. All methods were carried out in accordance with relevant guidelines and regulations. Confidentiality was also observed throughout the entire study. Authors did not have access to information that could identify individual participants during and after data collection thereby ensuring the anonymity of participants.

### Data analysis

Initial entry and organization of data were done using Microsoft Excel (version 2013). The data were cleaned and imported into STATA (StataCorp LLC, College Station, TX 77845, USA) statistical software version 15.1 for analysis. Descriptive statistics (frequency tables) were used. Data was tested for normality using Shapiro-Wilk Test, and Skewness and kurtosis Test for normality and found to be non-normally distributed. As such, the Wilcoxon Rank-Sum (a non-parametric equivalent of t-test among two groups) was used to compare biochemical and haematological parameters among participants based on renal outcome (dead or alive, dialysis or not). Based on that, predictors of renal disease outcomes among the children were established using bivariate and multivariate logistic regression analyses, and outcomes were presented with crude and adjusted odds ratios respectively. All analyses were done at a 95% confidence interval and p-values less than or equal to 0.05 were considered statistically significant.

## Results

The study involved 332 children (predominantly males [60.98%]) with a median age of 6 years [25th to 75th percentile (Q1 – Q3) being 3–9 years] and majority (44.27%) being five years or below (Table 1). The overall median duration of clinic visits was 2 months (Q1 – Q3 being 1–9 months) (Table 1).

**Table 1:** Demographic characteristics and duration of admission among study participants.

Variable	Number (n)	Percentage (%)
**Age Category (n=323); Median = 6.0 (3.0–9.0) years**		
0–5 years	143	44.27
5.1–10 years	127	39.32
10.1–15 years	53	16.41
**Sex (n=328)**		
Male	200	60.98
Female	128	39.02
**Duration of clinical visits (n=331); Median = 2.0 (1.0–9.0) months**		
1 month or less	158	47.73
Between 1 and 9 months	84	25.38
9 months and above	89	26.89

**Fig 1:**
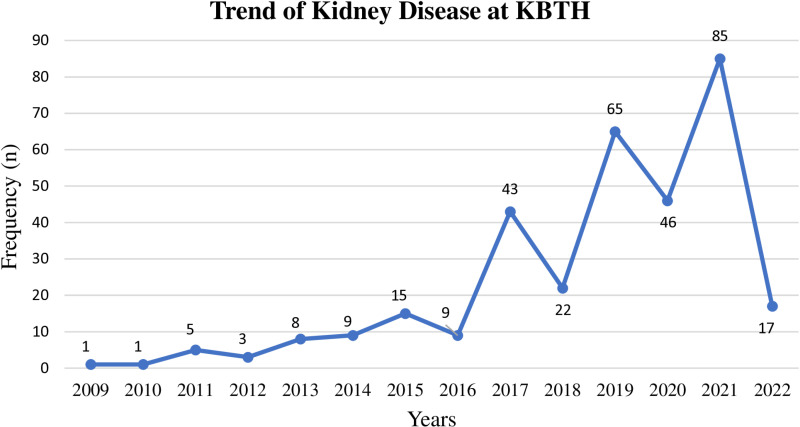
Trends of paediatric kidney disease among children at KBTH.

The most number of paediatric renal cases (n=85) was recorded in the year 2021 and the least number of cases were reported in 2009 (n=1) and 2010 (n=1) (Fig 1).

The main types of renal diseases among participants were nephrotic syndrome (42.47%), acute kidney injury (26.20%), and acute glomerulonephritis (14.16%). Other types of renal diseases identified included polycystic kidney disease (0.90%), lupus nephritis (0.60%), and others (ureterostomies, bladder calculus, and dilated ectopic renal pelvis) (1.81%) (Table 2). A mortality of 15.06% was observed among participants resulting mostly from AKI (6.33%), acute on chronic kidney disease (4.52%), and CKD (2.41%) while 7.83% underwent dialysis (Table 2). The majority of those who underwent dialysis (5.42%) had one to four sessions (Table 2).

**Table 2:** Types and major outcomes of renal diseases among study participants.

Variables	Frequency (n)	Percentage (%)
**Type of Renal Disease (n=332)**		
Nephrotic syndrome	141	42.47
Acute kidney injury	87	26.20
Acute glomerulonephritis	47	14.16
Acute on chronic kidney disease	20	6.02
Chronic kidney disease	14	4.22
Pyelonephritis (acute, left, etc.)	7	2.11
Hydronephrosis (fetal, bilateral, etc.)	5	1.51
Polycystic kidney disease	3	0.90
Lupus nephritis	2	0.60
Others (ureterostomies, bladder calculus and dilated ectopic renal pelvis)	6	1.81
**Outcome of Renal Disease**		
No Dialysis	306	92.17
Dialysis	26	7.83
*1 to 4 Sessions*	18	5.42
*5 sessions and above*	6	1.81
*Peritoneal Dialysis*	2	0.60
Dead	50	15.06
*Acute kidney injury*	21	6.33
*Acute on chronic kidney disease*	15	4.52
*Chronic kidney disease*	8	2.41
*Nephrotic syndrome*	4	1.20
*Acute glomerulonephritis*	1	0.30
*Bilateral hydronephrosis*	1	0.30

Table 3 shows the causes of renal diseases among participants. The major cause of nephrotic syndrome was idiopathic (unknown) causes (82.98%) while that of acute kidney injury was intravascular hemolysis secondary to malaria (41.38%) (Table 3).

**Table 3:** Causes of renal disease among study participants.

Variable	Number (n)	Percentage (%)
**Nephrotic Syndrome (n=141)**		
Idiopathic	117	82.98
Infection (*E. coli*, enterobacterial)	8	5.67
Congenital	4	2.84
Others (herbal medication intoxication, minimal change disease, encephalopathy, malaria, nephritis, renal impairment)	12	8.51
**Acute Kidney Injury (n=87)**		
Intravascular haemolysis secondary to	49	56.32
*Malaria*	36	41.38
*Sepsis*	6	6.90
*G6PD deficiency, General hemolysis*	4	4.60
*ABO incompatibility, Quinine Reaction*	3	3.45
Infection	9	10.34
*Streptococcal*	5	5.75
*Other bacterial infection (Klebsiella spp., Enterobacterial) and Urinary tract infection*	4	4.60
Dehydration	8	9.20
Idiopathic	8	9.20
Others (sickle cell disease, birth asphyxia, herbal medicine intoxication, anaemia, obstruction, gastroenteritis, Kawasaki disease, purpura fulminans)	13	14.94
**Other Renal Diseases (n=104)**		
Infection	47	45.19
*Streptococcal*	35	33.65
*Other bacterial infection (Klebsiella spp., Staphylococcus spp.)*	7	6.73
*Urinary tract infection, Human Immunodeficiency Virus, Endocarditis and Osteomyelitis*	5	4.81
Idiopathic	22	21.15
Hypertension	6	5.77
Congenital	5	4.81
Intravascular hemolysis 2 ֠ malaria	3	2.88
Others (dehydration, renal impairment, encephalopathy, post nephrotomy, schistosomal glomerulopathy, birth asphyxia, anaemia, obstruction, gastroenteritis, systemic lupus erythematosus, abnormal immune reaction)	21	20.19

2 ֠- secondary to

Table 4 describes the clinical presentation of the participants. The most frequently reported symptoms were swelling (76.96%), facial puffiness (39.94%), and fever (35.98%) whereas the least frequent symptoms included anorexia (3.05%), anuria (3.05%) and dyspnoea (1.52%).

**Table 4:** Clinical characteristics of study participants.

Variable	Number (n)	Percentage (%)
**Common Presenting Symptoms (n=328)		
Swelling (Body, pedal, scrotal, leg, generalized)	259	76.96
Facial Puffiness	131	39.94
Fever	118	35.98
Pains (Abdomen, joint, chest, knee, generalized)	94	28.66
Abdominal distension	77	23.48
Vomiting	76	23.17
Cough	76	23.17
Difficulty breathing	54	16.46
Haematuria	46	14.02
Skin rashes	45	13.72
Pale	33	10.06
Oliguria	31	9.45
Headache	27	8.23
Diarrhoea	21	6.40
Dysuria	14	4.27
Sore throat	13	3.96
Anorexia	10	3.05
Anuria	10	3.05
Dyspnoea	5	1.52

**Multiple responses applicable

Table 5 shows predictors of dialysis among participants who received dialysis. Controlling for urea and creatinine that are known predictors of dialysis, being a female (aOR 3.31; 95% CI 1.27–8.63, p= 0.01) and having acute on chronic kidney disease (aOR 10.19; 95% CI 1.04–100.05, p= 0.046) were significant predictors of dialysis among participants who received dialysis (Table 5).

**Table 5:** Predictors of dialysis among participants who received dialysis.

Variable	cOR	p-value	95% CI	aOR	p-value	95% CI
Sex (Male)	Ref	Ref	Ref	Ref	Ref	Ref
Female	**2.40**	**0.03**	**1.07–5.40**	**3.31**	**0.01**	**1.27–8.63**
Age Category (<5yrs)	Ref			–	–	–
5-10 years	1.98	0.149	0.78–4.50	–	–	–
>10 years	1.91	0.274	0.60–6.11	–	–	–
Haemoglobin (Normal: Hb>11.0g/dL)		Ref	Ref	Ref	Ref	Ref
Anaemia	**7.74**	**0.006**	**1.79–33.35**	3.22	0.153	0.65–15.99
Urea (Normal Urea: ≤7.8mmol/L)		Ref	Ref	Ref	Ref	Ref
High Urea	**17.95**	**<0.0001**	**4.17–77.31**	**–**	**–**	**–**
WBC count (≤15 x10^9^/L)		Ref	Ref	–	–	–
>15 x10^9^/L	0.66	0.415	–	–	–	–
Type of Kidney Disease (nephrotic syndrome)			Ref	Ref	Ref	Ref
Acute kidney injury	**34.95**	**0.001**	**4.54–269.22**	9.99	0.051	0.99–100.90
Acute on CKD	**36.81**	**0.001**	**4.35–311.44**	**10.19**	**0.046**	**1.04–100.05**

Creatinine had no odds due to co-linearity (all children who went for dialysis had high creatinine results). CKD = Chronic Kidney Disease, WBC = White Blood Cell count and Hb = Hemoglobin.

Having high WBC count (aOR 3.47, 95% CI 1.36–8.87, p = 0.009) and having acute on chronic kidney disease (aOR 47.30, 95% CI 3.57–626.54, p= 0.003) were significant predictors of death among participant (Table 6). There were over three times increased odds of death among participants with high WBC count compared to those with normal WBC count (aOR 3.47 ± 1.66, 95% CI 1.36–8.87, p = 0.009).

**Table 6:** Predictors of death among study participants.

Variable	cOR	p-value	95% CI	aOR	p-value	95% CI
Haemoglobin (Normal Hb: Hb>11.0g/dL)		Ref	Ref	Ref	Ref	Ref
Anaemia (Low Hb)	**3.16**	**0.003**	**1.47–6.75**	1.79	0.300	0.59–5.42
WBC Count (≤15 x10^9^/L)		Ref	Ref	Ref	Ref	Ref
>15 x10^9^/L	**3.22**	**<0.0001**	**1.74–5.97**	**3.47**	**0.009**	**1.36–8.87**
Urea (Normal Urea: ≤7.8mmol/L)		Ref	Ref	Ref	Ref	Ref
High Urea	**8.33**	**<0.0001**	**4.03–17.20**	2.61	0.197	0.61–11.21
Creatinine (Normal Creatinine: ≤100umol/L				Ref	Ref	Ref
High Creatinine	**7.58**	**<0.0001**	**3.94–14.61**	0.92	0.913	0.21–4.05
Cause of Kidney disease (Hypertension)		Ref	Ref	Ref	Ref	Ref
Idiopathic	3.05	0.095	0.82–11.28	3.32	0.193	0.54–20.29
Intravascular haemolysis	**6.45**	**0.006**	**1.73–24.04**	2.30	0.368	0.39–15.60
Infection	3.33	0.074	0.89–12.53	3.87	0.179	0.54–27.79
Other causes	**8.59**	**0.001**	**2.39–30.79**	6.35	0.072	0.87–47.25
Type of Kidney Disease (Acute Glomerulonephritis)			Ref	Ref	Ref	Ref
Nephrotic Syndrome	2.37	0.426	0.28–19.76	3.10	0.383	0.24–39.32
Acute Kidney Injury	**15.08**	**0.009**	**1.95–116.51**	5.38	0.161	0.51–56.58
Acute on CKD	**74.31**	**<0.0001**	**9.11–605.85**	**47.30**	**0.003**	**3.57–626.54**
Others	2.19	0.586	0.13–36.73	1.92	0.685	0.08–44.82

## Discussion

This study assessed the pattern, spectrum, outcome, and predictors of dialysis and death among children with renal disease at the Child Health Department of the KBTH, Accra, Ghana.

The majority of our study participants were found to be males (60.98%). This gender pattern is consistent with previous studies conducted by Wordui (2021) in Ghana (male: female ratio of 1.7:1), Obiagwu et al. (2019) in Nigeria (male-63.5%) and Anigilaje & Adesina (2019) in Nigeria (male-58.3%) [3,8,14]. These findings, therefore, stand to suggest that males are a higher risk group for paediatric renal diseases than females. The majority of the participants in our study were aged from 0 to 5 years (44.27%). This is also similar to what was reported by Anigilaje & Adesina (2019) and Mola & Damte (2016) as both reported their predominant age group to be less than 5 years of age (46% reported by each study) [3,5]. Our finding however differed from studies conducted by Yadav et al (2016) and Obiagwu et al (2019) who reported children aged 6–10 years (44.1%) and 10–15 years (45%) as the predominant population in their study respectively [6,14]. These variations could be due to differences in sample size, study design, and duration. It could also be due to variations in the rates of renal diseases among each year group.

In agreement with Wordui (2021) (NS-65.5%), Obiagwu et al (2019) (NS-33.6%) and Adedoyin et al (2012) (NS-42.1%), our study also reported nephrotic syndrome (NS) (42.47%) as the most common renal disease [8,9,14]. This finding differed from studies conducted in Nigeria and Nepal by Anigilaje & Adesina (2019), Ocheke et al (2010), and Yadav et al (2016) who reported [urinary tract infection (30.7%) and acute kidney injury(30.7%)], acute glomerulonephritis (37.7%) and acute glomerulonephritis (37.7%) as the most common renal diseases respectively [3,6,7]. Although the reasons for the disparity in the spectrum of renal diseases are unclear, they may be linked to variations in environmental risk factors for renal diseases, clinical and laboratory capacities to diagnose renal diseases, referral patterns, research duration, and sample size. Acute kidney injury (26.20%) was the second most frequent renal disease in this study. This directly correlates with the high AKI incidence (31.02%) reported by Antwi et al. (2015) in Kumasi [10]. This indicates a significant burden of acute kidney injury among hospitalized children in Ghana.

The causes of nephrotic syndrome (NS) among children at KBTH had idiopathic (unknown) causes emerging as the major cause (82.98%). This increased number of idiopathic (unknown) causes of NS was also reported by Ibeneme et al., 2021 (80%) and Ladapo et al, 2014 (90.2%) in similar studies [4,13]. The reason for this increased prevalence of idiopathic causes remains unclear but may be attributed to late referral or presentation and the unavailability of diagnostic services in hospitals as reported in Sub-Saharan African settings making it difficult the evaluate the etiology of the NS. In contrast to the findings of Okyere et al (2020), Kaze et al (2015) and Antwi et al (2015) which found sepsis (48.6%), renal parenchymal disease (58.7%), hemoglobinuria from different aetiologies (15.0%) as the major cause of AKI, our study projected intravascular hemolysis principally secondary to malaria (41.38%) as the major cause of AKI [10,12,20]. These variations may be due to differences in the population setting, as well as the diagnostic resources employed.

This study reported a variety of clinical presentations (symptoms) among children with renal disease. Swelling (76.96%), facial puffiness (39.94%), fever (35.98%), pains (28.66%), and abdominal distension (23.48%) were the most common symptoms among participants. Similar clinical presentations including fever, swelling, vomiting, abdominal distension, pain, facial swelling, and anuria among others have also been reported in similar studies by Anigilaje & Adesina (2019), Halle et al (2017) and Yadav et al (2016) [3,6,16]. However, the variety of clinical presentations observed among children with renal diseases could be due to the stage of disease at presentation and underlying etiologies.

According to our study, 7.83% of the participants underwent dialysis. This finding is lower than the 12.14% reported by Antwi et al (2015) in Kumasi, Ghana [10]. Financial difficulty in accessing dialysis services could account for our observation. Furthermore, our study revealed being a female and having acute on chronic kidney disease as significant predictors of dialysis among participants who received dialysis. Our study also revealed a death rate of 15.06% which is higher than the 14.1%, 14.4%, 5.1%, and 5% death rate reported among children with renal diseases [2–4,6]. The high mortality rate found in our study could be attributed to several factors, including the severity of the renal disease at presentation in the hospital, limitations in care, the impact of the underlying disease, and financial constraints, especially for those requiring this intervention. This death rate resulted mostly from AKI (6.33%), acute on chronic kidney disease (4.52%), and CKD (2.41%). This is similar to studies by Anigilaje & Adesina (2019) and Onifade (2003) who reported AKI, CKD, and acute glomerulonephritis to be majorly associated with death among children with renal diseases [3,15]. Having a high WBC count and acute on chronic kidney disease were found to be significant predictors of death among children with renal disease, and thus these parameters should be closely monitored during their treatment. This observed poor prognosis of children with acute on chronic kidney disease could be because these children may have already compromised renal reserve and function long-term from chronic kidney disease and therefore when acute injury subsequently develops, the kidneys may have a more limited ability to recover and repair due to the underlying chronic impairment. In contrast, Halle et al (2017) reported young age, presence of a coma, use of herbal concoctions, and acute pulmonary edema as factors associated with mortality among children with renal disease [16].

Our study has several limitations. To begin with, our study was retrospective, so biases such as patient selection and access to information are unavoidable, as some children may have been inadvertently excluded from the register. This can be observed with the few numbers of cases recorded from 2009 to 2012. Additionally, our study could not account for the total number of participants who needed dialysis, those who could not get access to dialysis, and the number of deaths associated with dialysis. Also, the study did not capture data on congenital anomalies of the kidney and urinary tract (CAKUT), which are known to be a significant contributor to paediatric renal diseases. The absence of this information limits the comprehensive understanding of the etiological factors and patterns of renal diseases in the study population. Furthermore, the diagnosis of renal diseases was not exclusively made by the data collectors, but rather relied on the clinical assessments and documentation by the attending healthcare providers. This may introduce some variability in the strict application of the operational definitions and diagnostic criteria. Finally, the study was unable to determine the severity of kidney disease at the time of presentation, which could have provided valuable insights into the progression and outcomes of the various renal conditions.

## Conclusion

This study provides insight into the burden of paediatric renal diseases, their relative occurrence, clinical profile, causes, outcomes, and predictors of death and dialysis with nephrotic syndrome and acute kidney injury found to be most prevalent. Infectious agents and various conditions resulting in intravascular hemolysis contribute to the incidence of paediatric renal diseases in Ghana and must be adequately examined and treated to prevent them from progressing into various forms of renal diseases. This study also heightens the need for routine screening for renal diseases in children, the development of paediatric renal services with access to subsidized renal healthcare services to improve the health status, well-being, and early detection of paediatric renal diseases.

## Supporting information

S1 File Dataset. (XLSX)
